# Cecal Volvulus Diagnosed with a Whirl Sign: A Case Report

**DOI:** 10.21980/J8XM05

**Published:** 2020-10-15

**Authors:** Gregory K Sun, Brian Walsh

**Affiliations:** * Morristown Medical Center, Department of Emergency Medicine, Morristown, NJ

## Abstract

Cecal volvulus is responsible for about 1.9% of all colonic obstructions in the United States.^1^ Common physical exam findings are abdominal distension with generalized abdominal tenderness; however, the presentation can vary.^2^ A 64-year-old female presented with right upper quadrant abdominal pain associated with nausea and vomiting. On physical examination, Murphy sign was present. A comprehensive ultrasound was negative for cholecystitis. The diagnosis of cecal volvulus was made using computed tomography (CT), which demonstrated a “whirl sign.” Surgery was consulted and emergently took the patient to the OR for a detorsion and right hemicolectomy. The patient made a full recovery with return of normal bowel function on post-op day 3. Additionally, no further adverse effects reported on follow-up. This case report further emphasizes the importance of maintaining a wide differential for patients with abdominal pain regardless of the location in the abdomen. In addition, when suspecting a bowel obstruction, the “whirl sign” is a visual diagnosis suggestive of a cecal volvulus requiring emergent surgical consultation.

**Topics:**

Cecal volvulus, abdominal pain, whirl sign, right upper quadrant, CT.

**Figure:**
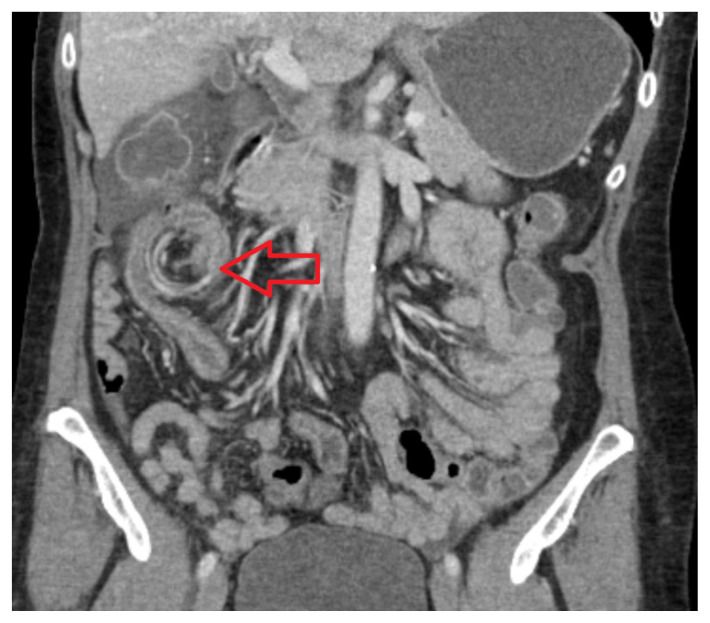

**Figure:**
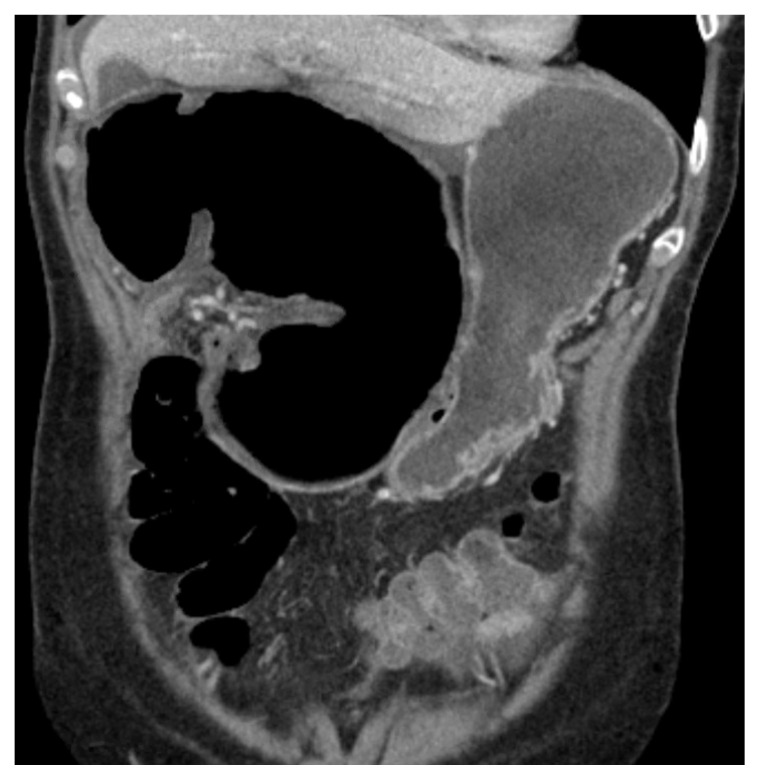

## Introduction

Abdominal pain is one of the most common complaints in an emergency department with a large number of causes.^3^ Cecal volvulus is a rare cause, found to be associated with long distance runners^4^ and causes 1.9% of all small bowel obstructions.^1^ Most patients present with generalized abdominal distension, generalized pain, vomiting and nausea.^2^ Diagnostically, “whirl sign” is a rare CT finding, which makes this case report notable. The treatment for a small bowel obstruction usually begins with gastric decompression which can be futile if a volvulus is causing the obstruction.^5^ Surgery is almost always indicated in the case of volvulus.^2^ If there is a delay in surgery, ischemic bowel can result leading to a septic abdomen and death.^5^

## Presenting concerns and clinical findings

The patient was a 64-year-old female who presented with severe, sharp epigastric and right upper quadrant pain described as “cramps” for the last 12 hours. This pain was associated with nausea and two episodes of emesis after ingestion of food. She had no surgical history but reported a family history of cholelithiasis. Of note, her social history revealed that she exercised regularly, including casually running about once a week. Pertinent negatives included no fevers, chest pain, diarrhea, melena, or hematochezia. The patient’s abdominal exam showed a positive Murphy sign with right upper quadrant tenderness. No abdominal distension or rebound tenderness were noted.

## Significant findings

The CT image demonstrates a “whirl sign” (red arrow) which is indicative of a volvulus.^6^ This image occurs when bowel, mesentery and vasculature rotate around a transition point causing an image similar to a hurricane on a weather map.^6^ When seen on a CT scan, a whirl sign suggests a high likelihood of either a closed loop bowel obstruction or volvulus in the cecum, sigmoid or midgut.^7^ In any of the cases, seeing a whirl sign strongly increases the need for emergent surgical management.^5^

## Patient course

Based on the presenting symptoms, a comprehensive abdominal ultrasound was initially performed to assess for concern for cholecystitis but was negative. The patient’s white blood count, hemoglobin and lactate were found within normal. During the evaluation, patient reported symptoms worsened with continued significant abdominal tenderness despite administration of 15 mg Ketorolac IV and 4mg Morphine IV. An abdominal x-ray was also ordered to evaluate free air and perforation. The x-ray showed air-fluid levels in the lateral view, leading to concerns of a small bowel obstruction. A CT scan of the abdomen with PO contrast was ordered. Due to the pain, the patient could only ingest 500mL of the PO contrast. However, this did not affect the image quality. On emergency provider review of the CT scan, a whirl sign was noted concerning for obstruction as seen on figure 1. Radiology confirmed the presence of a whirl sign due to suspicion of cecal volvulus as seen on figure 2. Surgery was consulted for management. The patient was sent emergently to the OR and underwent laparoscopic detorsion of the volvulus followed by a right hemicolectomy. On hospital day 3, the patient regained normal bowel function, and there were no reported complications in post-operative period. She was discharged on pain medication and instructed to follow up in 10 days. Per follow-up notes from the surgical team, the patient has had no further complications or adverse events on subsequent outpatient visits.

## Discussion

Abdominal pain is a common complaint in the Emergency Department and the location of pain can allow us to tailor our differential.^3^ The patient in this case had right upper quadrant pain and tenderness, and our top differential was cholecystitis based on the history and exam. Our initial workup was negative, but because of continued suspicion for serious causes of her symptoms, we added additional imaging. The additional workup ultimately led to the final diagnosis of cecal volvulus. Localized abdominal pain is an atypical presentation for cecal volvulus, and this case report emphasizes the need for a broad differential guided by repeat serial abdominal exams.

A bowel obstruction secondary to a cecal volvulus was diagnosed when the CT scan revealed a whirl sign. The whirl sign was first documented in 1993 for a malrotation of the midgut.^7^. It has since also been associated with a volvulus or small bowel obstruction requiring surgical intervention.^2^ The statistical values vary in regard to the whirl sign predicting a volvulus.^8^ A large group study in oncology patients demonstrated a sensitivity of 45%, specificity of 98%, positive predictive value 22%, and negative predictive value of 99%.^8^ While a whirl sign does not always predict a volvulus, the sign does seem to indicate the need for surgical intervention. Patients were found to be 25.3 times more likely to be treated with surgery in the presence of an SBO (small bowel obstruction) when a whirl sign was found.^5^ The whirl sign is a significant prognostic indicator, and while it is not always specific for volvulus, it does indicate the need for emergent surgery.^9^

Our case demonstrates a wide differential is needed when examining a patient with an acute abdomen. Specifically, cecal volvulus can present with a variety of symptoms. Continuing to reevaluate the patient, recognizing when medical therapies are not adequate, and changing the workup to reflect new diagnoses is important for an emergency medicine clinician. When seen on CT scan, a whirl sign suggests a diagnosis of a closed-loop obstruction or volvulus in the cecum, sigmoid, or midgut, and indicates the need for surgical intervention.

## Supplementary Information




