# Supplementary figures and images for: Cecal Volvulus Diagnosed with a Whirl Sign: A Case Report
Source: J Educ Teach Emerg Med. 2020 Oct 15;5(4):V22–4. doi: 10.21980/J8XM05 (PMC10332527; doi:10.21980/J8XM05)

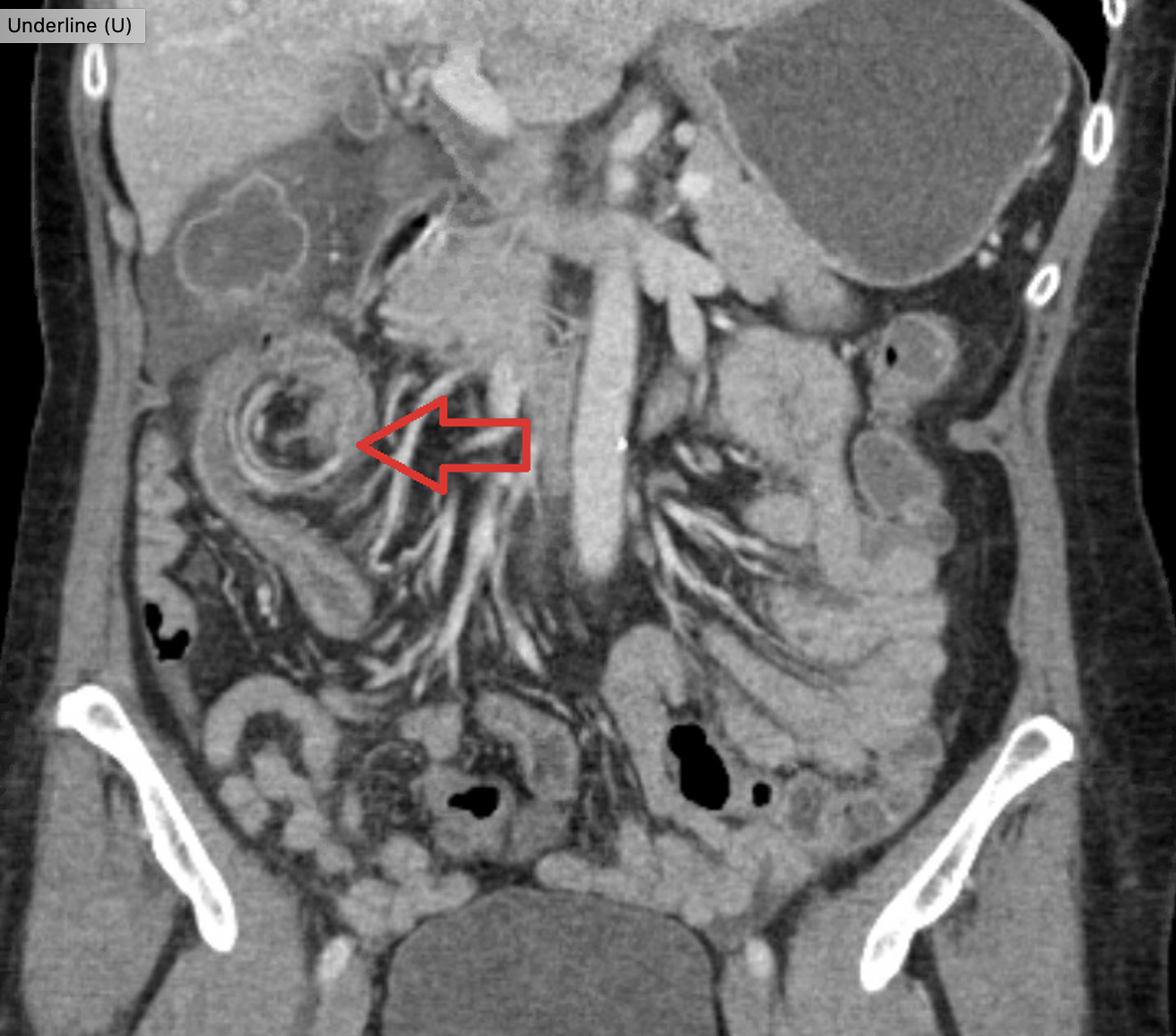

Supplement: Supplementary file 1 [file jetem-5-4-v22-supp1.jpeg]

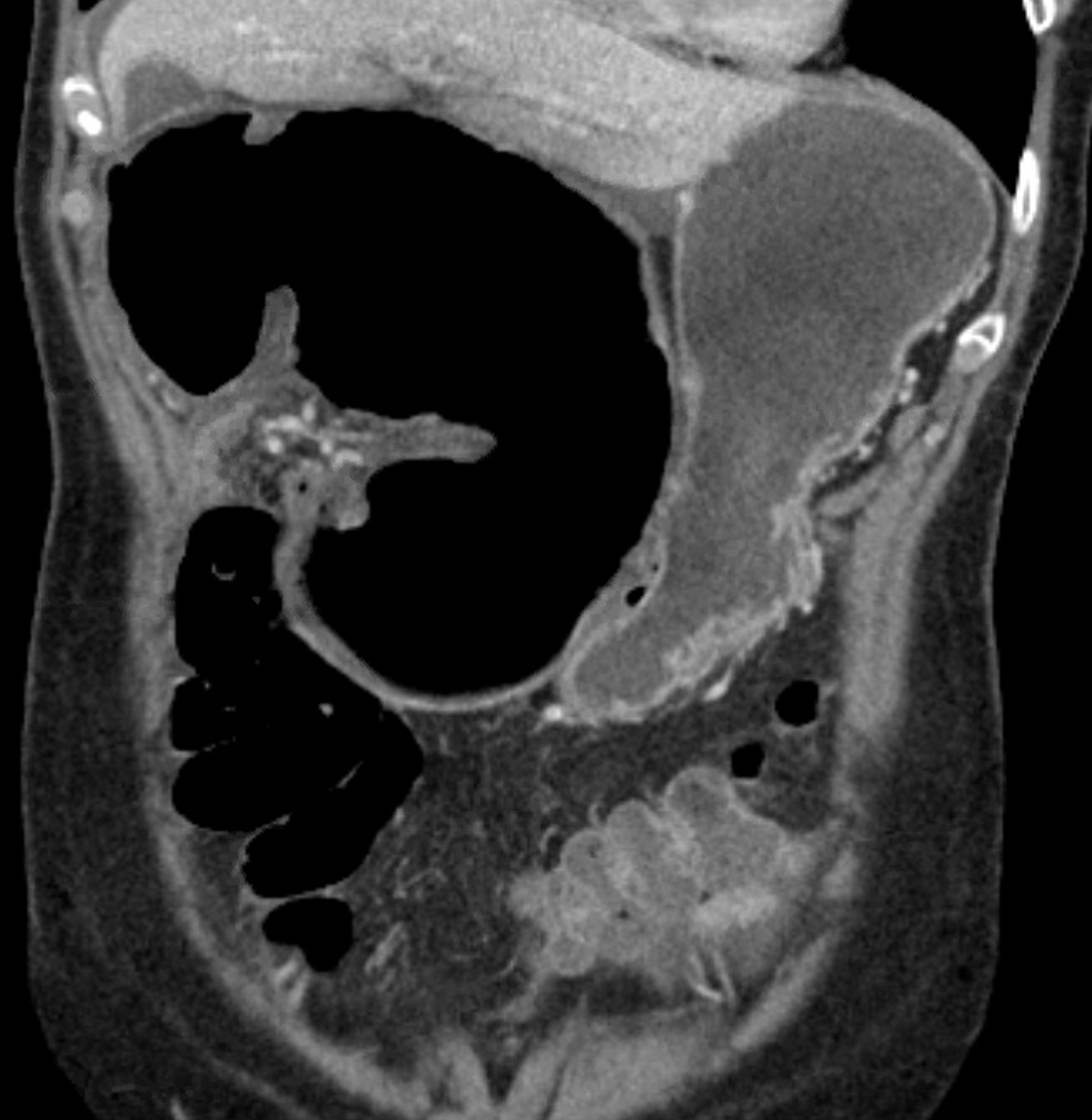

Supplement: Supplementary file 2 [file jetem-5-4-v22-supp2.jpeg]
